# The Role of Selected Psychological Factors in Healthy-Sustainable Food Consumption Behaviors during the COVID-19 Pandemic

**DOI:** 10.3390/foods11131944

**Published:** 2022-06-29

**Authors:** Francesco Zanatta, Silvia Mari, Roberta Adorni, Massimo Labra, Raffaele Matacena, Mariangela Zenga, Marco D’Addario

**Affiliations:** 1 Department of Psychology, University of Milan-Bicocca, 20126 Milan, Italy; silvia.mari@unimib.it (S.M.); roberta.adorni1@unimib.it (R.A.); marco.daddario@unimib.it (M.D.); 2 BEST4Food-Bicocca Center of Science and Technology for Food, University of Milan-Bicocca, 20126 Milan, Italy; massimo.labra@unimib.it (M.L.); raffaele.matacena@unimib.it (R.M.); 3 Department of Biotechnologies and Biosciences, University of Milan-Bicocca, 20126 Milan, Italy; 4 Department of Statistics and Quantitative Methods, University of Milan-Bicocca, 20126 Milan, Italy; mariangela.zenga@unimib.it

**Keywords:** SARS-CoV-2, pandemic, healthy-sustainable diets, health behaviors, anxiety, depression, stress, subjective well-being, lockdown, nutrition

## Abstract

The COVID-19 pandemic and the consequent precautions and dispositions adopted have triggered substantial changes in daily health-related behaviors, including food consumption habits. The psychological impact of the pandemic has been considered one of the factors affecting this transition and requiring consideration when targeting healthy-sustainable behavior preservation. The present study describes the results of a survey conducted on a convenience sample of Italian residents (*n* = 2272) during the first phases of pandemic. The aim was to explore the daily nutritional choices and behaviors and their transformations that occurred along with the associations with psychological factors (i.e., subjective well-being, and depression, anxiety and stress symptoms). An indicator for healthy-sustainable transition (HST index) was constructed and revealed diffused transformation in dietary habits, with a large segment of the sample adopting healthier and more sustainable dietary behaviors and others showing reduced healthy-sustainable food choices. Informative relationships with the psychological variables were then found from the correlational and regression analyses. Lower levels of anxiety, depression and stress symptomatology and higher perceived subjective well-being were significantly associated with healthier-sustainable food consumption behaviors. These findings shed light on the crucial areas to be considered in future institutional interventions, ultimately ensuring favorable conditions for both healthy diet behaviors and sustainable food consumption choices.

## 1. Introduction

The Severe Acute Respiratory Syndrome-Coronavirus-2 (SARS-CoV-2) pandemic outbreak has brought considerable changes to the lives of many individuals. In response to its rapid spread, immediate precautions (e.g., social-distancing, activity shutdowns, hygienic measures) were adopted. In particular, in March 2020, Italy was the European country reporting the highest number of cases and deaths [1], consequently requiring very stringent and rigid dispositions in order to safeguard people’s health. During the earlier phases of the pandemic, Italian citizens were housebound and only a few activities were allowed (i.e., going to work, going to the hospital, and grocery shopping). As for Italy and most countries worldwide, not only has this event constituted an unprecedented public health crisis, but it has also been a challenging occurrence that has triggered significant modifications in people’s daily routines and habits. Concurrently, besides the consequent and increasing exposure to health concerns, the worldwide community has found itself having to deal with lifestyle and health-related behavior adaptation. For instance, profound effects were observed on food consumption and dietary intake. Due to restricted store opening hours, reduced availability of goods, as well as more time spent at home, significant effects on food purchasing and preparation were observed, thereby affecting diet quality [2]. This situation has led to recent systematic reviews and meta-analyses that have reported informative and mixed evidence, across different countries, age groups, and general and clinical populations. Gonzales-Monroy et al. [3] showed, for example, a worsening of diet between the pre-pandemic and pandemic period with an increased preference to consume snacks, sweets, and ultra-processed foods rather than vegetables, fruits, and fresh food. In contrast, other research found an increase in adherence to the Mediterranean diet [4], indicating that the pandemic did not necessarily have a negative impact on eating behaviors.

Contextually, nutrition has increasingly gained attention, occupying a central position in individual health and, more broadly, in socio-environmental sustainability issues [5]. On the one hand, it is well recognized that the adoption of healthy eating behaviors and of a varied nutritional regimen is crucial when aiming to maintain good health. Accordingly, nutritional status was highlighted as a factor predicting health outcomes of patients with COVID-19 [6], suggesting that worse profiles are more likely to develop severe medical conditions [7,8]. In support of this, prior research has oppositely shown that healthy diets have the potential to benefit the immune system, sensibly decreasing COVID-19 vulnerability [9]. On the other hand, nutrition in the pandemic era has become a central theme towards sustainable habits and conduct. There is a growing awareness that the maintenance of a diet that is both healthy and sustainable will be increasingly difficult to achieve. This is because, in present times, the food economy is based on a production-consumption model that, especially during the pandemic, has turned out to be unsustainable [10]. This paradigm encompasses a global food trade deeply connected to an industrial agricultural and urbanization development model that has been threatening to worldwide ecological and cultural biodiversity. Therefore, today more than ever, reforming the present paradigm starting from dietary pattern sustainability has become an urgent challenge. In 2019, the Food and Agriculture Organization of the United Nations (FAO) recognized sustainable diets to be crucial in promoting all dimensions of individual health and well-being. This allowed the optimization of the environmental costs of food production and the consumption and adaptability to local social, cultural and economic contexts. At the country level, the adoption of such guiding principles that take a holistic approach to diet not only embodies a valuable solution, but also represents a precious contribution towards the achievement of the well-known sustainable development goals (SDGs) [11].

Along these lines, there is a growing need for analysing eating behaviors not exclusively on an individual-centric level, but also by including their relationships with external and contextual factors (economic, social, and cultural) [12]. In this light, the pandemic has represented an opportunity to explore, under unprecedented circumstances, the propensity of individuals to make more or less healthy and sustainable food choices, making it possible to advance informative insights into future directions and transformations. As highlighted in a prior work [13], the COVID-19 crisis allowed the identification of strategic areas of institutional intervention on which to focus to ensure more sustainable food production and consumption. The survey, conducted during the earlier phases of the first nationwide lockdown in Italy, described healthy-sustainable food consumption transformations across different generational cohorts, places of residency, educational levels, subjective social status, socio-economic condition, working conditions, and household compositions.

To date, what has been scarcely addressed, however, is the relationship between the adoption of sustainable food choices and psychological factors. More broadly, diverse studies have exclusively explored the associations of an individual’s psychological responses to the COVID-19 pandemic with lifestyle and health behavior changes [14,15,16,17], adopting, however, an individual-centric framework. As specifically concerns the changes in dietary behaviors, recent studies showed that stay-at-home restrictions, including teleworking and as social isolation, in some cases, to have triggered stress, anxiety and mood disorders throughout the population [18], ultimately affecting food choices. In other cases, it must be noted that the COVID-19 pandemic has represented an opportunity for positive changes in dietary habits [19,20,21,22]. These improvements have been attributed, for example, to having more free time to cook healthy meals, to a pre-pandemic more advantageous socio-economic status, or also to the motivation to improve health in order to reduce the fear of higher susceptibility to COVID-19 symptomatology [13,23].

In light of this background, the present study aims to provide further insight into the impact of the COVID-19 pandemic during its early phase (March–May 2020) on food consumption behaviors in a convenience sample of Italian residents. More specifically, the study has a twofold purpose: (i) to analyse dietary changes taking into account both the healthiness and the sustainability of food-related choices, and (ii) to further explore their associations with the psychological responses to the pandemic, namely pandemic-related depression, anxiety, and stress symptoms, and the perceived subjective well-being during the lockdown. Based on the aforementioned prior works [14,15,16,17,18,19,20,21,22], we hypothesize that, during the pandemic, depression, anxiety, and stress symptoms may have contributed to negatively affect healthy-sustainable food choices, while higher subjective well-being may have increased the tendency to adopt healthier and more sustainable eating behaviors.

## 2. Materials and Methods

The present study is part of a broader research project launched by the BEST4Food (www.bestforfood.unimib.it) interdepartmental center of the University of Milano-Bicocca (Milan, Italy). The general aim of this project was to investigate the psychological, social and sustainability correlates of individuals’ dietary behaviors throughout the emergency period of the COVID-19 pandemic. More broadly, the project was driven by the willingness to better understand the reactions of individuals to COVID-19 with the aim to obtain strategies and solutions to implement for a healthy-sustainable post-pandemic food system. The study was conducted in accordance with the guidelines of the Declaration of Helsinki and approved by the Ethics Committee of the Department of Psychology of the University of Milano-Bicocca (Protocol RM-2020-297, 13 May 2020).

### 2.1. Study Design, Procedures, and Participants

The current work is positioned in the first stage of the broader research project. Following the earlier phase of the nationwide lockdown (9 March to 4 May 2020) [24], from May to June 2020 an online survey was administered to 3630 Italian residents. Data were collected employing Qualtrics Software (Qualtrics, Version April 2020; Qualtrics, Provo, UT, USA). Participants were invited to participate using an online link posted on the official communication channels of the university (webpage, newsletter and social media) and through snowball personal communications of the researchers involved in the study. Informed consent to participate in the survey and data treatment were obtained from all respondents. Of the total number of surveys administered, 2272 complete responses were recorded (62.6% response rate). Regarding the characteristics of the final sample (Table 1), the mean age was 38.7 ± 14.0 years (range: 18–90), most were female (73.3%) and had graduated (42.8%). Moreover, during the lockdown the majority of participants declared to have worked from home (59.6%), and to have lived with others (two or more people, 88.3%) predominantly in the Lombardy region (67.9%). In conclusion, most of the cases reported that the COVID-19 crisis had no effect on the household economic condition (54.3%) or that it was slightly detrimental (29.0%).

### 2.2. Measures

The online survey included a questionnaire designed to collect socio-demographic and living conditions information, usual dietary regimen and habits along with self-reported changes in the practices related to food consumption and preparation, the psychological condition (i.e., subjective well-being, anxiety, depression and stress symptoms) under lockdown, information about health status (e.g., weight management, physical activity), and opinions, attitudes and future intentions towards food- and sustainability-related issues. Differently from our previous work [13], which accurately described the lockdown-related changes in dietary and cooking habits along with the socio-demographic and housing factors underlying a healthy-sustainable transition, the present work mainly focused on the dietary habit changes during the earlier phases of pandemic and their associations with the psychological factors evaluated. 

#### 2.2.1. Psychological Variables

The psychological assessment included validated scales and questionnaires measuring perceived subjective well-being, and the anxiety, depression and stress symptoms perceived during the last week. 

*MacArthur Scale*. This allows the evaluation of the subjective social status by a pictorial format measure represented by a 10-rung social ladder on which respondents have to indicate their socio-economic status in comparison to others in society based on income, educational level and occupation [25]. According to prior works within the psychological and health sciences fields [26,27], this scale allowed for the capturing of individuals’ social standing according to their own perspective and subjectivity across socio-economic status components, ultimately contributing to clarifying the connection underlying objective and subjective socio-economic status and psychological well-being. For the present study, the MacArthur Scale was therefore included to evaluate perceived subjective well-being, with higher scores indicating the perception of more positive profiles.

*Depression Anxiety Stress Scale (DASS).* The DASS consists of 21 items divided into three subscales, evaluating depression, anxiety, and stress symptoms that occurred in the past week. Examples of items (presented in the form of statement) are: “I found it hard to wind down”, “I was aware of dryness of my mouth”, and “I could not seem to experience any positive feeling at all”. Respondents were asked to rate how many of each item applied to them on a 4-point Likert scale (0 = did not apply to me at all; 3 = applied to me very much, or most of the time). Higher scores in all three subscales reflected more severe symptomatology. More specifically, subscale scores on symptom severity can be categorized as normal (Depression: 0–9; Anxiety: 0–7; Stress: 0–14), mild (Depression: 10–13; Anxiety: 8–9; Stress: 15–18), moderate (Depression: 14–20; Anxiety: 10–14; Stress: 19–25), severe (Depression: 21–27; Anxiety: 15–19; Stress: 19–25), and extremely severe (Depression: >28; Anxiety: >20; Stress: >34). The DASS has been previously validated in the Italian population, showing satisfactory levels of internal consistency [28] and, so far, it has proved to be a reliable tool for the evaluation of the psychological response to the COVID-19 crisis [29]. For the present study, Cronbach’s Alpha coefficients were calculated to test scale internal consistency [30]. Satisfactory reliability scores for each subscale were observed (Depression: α = 0.867; Anxiety: α = 0.798; Stress: α = 0.874).

#### 2.2.2. Healthy-Sustainable Food Consumption Behavior

**Figure 1:**
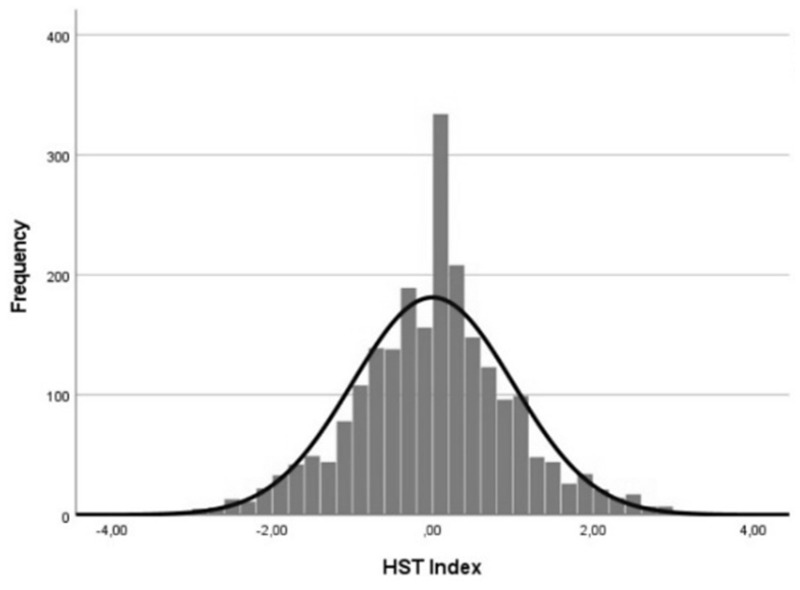
Distribution of the HST index scores in the sample.

Food consumption behaviors were assessed in terms of the usual dietary regime and of self-reported eating habit changes during the pandemic. First, participants were asked to indicate if they usually adopted an omnivore or pescatarian, vegetarian, vegan diet, or if they did not consume red meat exclusively. Then, to evaluate whether the consumer’s habits of food changed during the lockdown, a single question composed of eleven items was designed. These included food groups adapted from the EATLancet Commission’s guidelines for a planetary healthy-sustainable diet [31]. These guidelines consist of indications of consumption for different food categories within which it is possible to have a concurrent sustainable (i.e., low environmental impact) and healthy (i.e., able to prevent diet-related non-communicable diseases and mortality) diet. Accordingly, the EATLancet diet is mainly plant based, limiting the content of animal-based foods, saturated fats, and sugar, and emphasizing whole grains content, vegetables, legumes, fruit, and nuts. Based on this reference, two sub-groups were created, namely the sustainable foods (vegetable-based dishes, legumes, whole grain cereals, nuts and seeds, fresh fruit) and unsustainable foods (carb-based dishes, meat-based dishes, dairy products, sweets and desserts, alcoholic and sugary beverages) groups. Participants were asked to indicate on a 4-point Likert scale how often they used to consume each food during the lockdown in comparison to their prior ordinary habits (1 = *neither before pandemic nor during lockdown*; 2 = *less frequently than before*; 3 = *as usual, lockdown had no effect*; 4 = *more frequently than before*). Based on this data, a healthy-sustainable transition (HST) index was constructed to obtain an aggregate score for participants’ dietary transformations indicating whether and to what degree they represent a transition towards a more or less healthy-sustainable consumption model. To obtain a latent construct explaining food groups frequency results, the dimensionality of the data was reduced by identifying one or more factors that summarize the available items. Since foods were grouped in ordered categorical items, the categorical principal component analysis (CatPCA) [32] was applied. The categories of variables were assigned numeric values through optimal scaling [33,34], which allows for the transforming of categories of variables with ordinal levels into numeric value variables. This process ensured that, after replacing the category labels with category quantifications, as much as possible of the variance in the quantified variables was explained [33]. Goodness of fit evaluation was based on the total variance explained in the transformed variable (VAF) on the corresponding percentage (PVAF), and on a generalized version of Cronbach’s alpha [35,36]. The results generated from the whole procedure are presented in detail as supplementary material (see Tables S1–S6). Through the application of the CatPCA, the original categories of food frequency items were aggregated into three categories, namely “Less than before”, “Never or equal to before”, and “More than before”. The same procedure was then applied to the two food groups (sustainable and unsustainable foods) separately as well, thereby creating two independent indexes that reflected the changes in consumption of sustainable food (CCSF) and those in non-sustainable food (CCNSF). Finally, a PCA was applied to summarize the CCSF and CCNSF into a single indicator of healthy-sustainable transition of dietary behavior (HST index), in which the more the value is negative, the more the change reflects a transition towards less healthy and sustainable food choices, whereas the more the value is positive, the more the change corresponds to a transition towards healthier and more sustainable food choices. Figure 1 shows the final HST index calculated from the data collected from the study sample. Its scores ranged from −3.85 to 3.88 and appeared normally distributed (Skewness: 0.51 ± 0.51; Kurtosis: 0.82 ± 0.1), reflecting the diffused transformation of dietary habits, with a large segment of the population adopting healthier and more sustainable dietary behaviors and others showing reduced healthy-sustainable food choices. 

### 2.3. Statistical Analyses

Of the final sample, descriptive statistics were conducted on the socio-demographic, household, and clinical characteristics (i.e., generational cohorts, gender, place of residency, educational level, effects of COVID-19 pandemic on household economic conditions, working conditions under lockdown, household composition, and BMI, and scores were categorized according to the WHO guidelines and recommendations for a healthy lifestyle in the adult general population [37]), the usual dietary regime, the self-reported food consumption behavior changes during the lockdown (within both sustainable and unsustainable food groups), and the psychological responses to the pandemic. To analyze the relationship between healthy-sustainable dietary behavior change and the socio-demographic, clinical, and psychological characteristics, an independent samples *t*-test and one-way ANOVA was carried out, defining the HST index as a dependent factor. Between-groups differences were then estimated through post-hoc multiple comparisons procedures (LSD test). To investigate the associations of the HST index with the psychological variables more deeply, a correlational analysis was conducted along with the calculation of Pearson’s *r* coefficients. Furthermore, controlling for the socio-demographic, household, and clinical characteristics (i.e., age, gender, educational level, household economic condition, working condition under lockdown, household composition, and BMI) of the study sample, a multiple linear regression model was tested to estimate the simultaneous impact of the psychological variables (i.e., MacArthur Scale, DASS) on the HST index. From the regression analysis, R^2^ and *F* test values were computed for the explained variance and model fit, respectively. The analyses were carried out by means of the Statistical Package for Social Sciences (SPSS, version 27.0, IBM Corp, Armonk, NY, USA) software. All statistical tests were two-tailed and a *p*-value ≤ 0.05 was considered as statistically significant. 

## 3. Results

### 3.1. Changes in Dietary Habits 

Regarding the usual dietary habits before the COVID-19 pandemic outbreak, participants reported to be mostly omnivores (81%) with the rest of respondents declaring to adopt pescatarian, vegetarian or vegan diets (9.8%) or to consume no red meat (9.2%). Informative results on healthy-sustainable food consumption behavior changes were then observed. Overall, a transformation of dietary habits emerged, with a large portion of the samples reporting to have adopted healthier and more sustainable behaviors, while a proportioned segment of the sample declared to have worsened them. Investigating the changes in greater detail within both the sustainable and unsustainable food groups (Table 2), it is demonstrated that, for almost each food, the majority of participants have not changed their habits during the lockdown, while the remaining segment of the sample has shown higher or (in smaller percentages) lower consumption. A different sample configuration was particularly observed concerning the consumption of sweets and desserts, with most participants (47.0%) declaring to have increased their consumption when they were housebound.

Multiple comparisons on the HST index and the socio-demographic, clinical, and psychological variables were conducted. A significant difference on the HST index emerged for gender (t_(2267)_ = 1.440, *p* = 0.013), with men (0.05 ± 0.96) showing a significantly more positive transition towards healthy-sustainable dietary habits than women (−0.02 ± 1.01). Moreover, significant effects of the generational cohorts (F_(5,2266)_ = 2.751, *p* = 0.017), working condition (F_(3,2268)_ = 3.159, *p* = 0.024), and BMI (F_(3,2267)_ = 7.263, *p* < 0.001) were also observed. Between-group mean scores differences are presented in detail as supplementary material, and showed that younger participants (*p* = 0.002), those working from home (*p* = 0.003), and participants with lower BMI scores (*p* < 0.001) had a significantly more positive healthy-sustainable eating behavior change. Significant effects of the educational level, and household economic condition and household composition were not found. Table 3 specifically shows the comparisons with the psychological characteristics of the sample. Significant main effects of subjective well-being (F_(4,2258)_ = 2.400, *p* = 0.048), depression (F_(4,2106)_ = 3.355, *p* = 0.010) and stress (F_(4,1986)_ = 2.598, *p* = 0.035) symptoms were found. In particular, LSD post-hoc test procedures showed that HST index mean scores were significantly lower in participants with severe stress than in those reporting no (*p* = 0.009) or mild (*p* = 0.014) symptoms. Similarly, significantly lower mean scores were found in participants with severe (*p* = 0.010) and extremely severe (*p* = 0.012) depression symptoms, when compared to those reporting normal scores. Post-hoc comparisons on the HST index scores and the subjective well-being provided no significant results.

### 3.2. Associations between HST Index and the Psychological Variables

The inter-relationships among healthy-sustainable food consumption behaviors and the psychological variables were explored by means of correlational and regression analyses. From the correlational analysis (Table 4), it was found that the HST index significantly negatively correlated to anxiety (r = −0.053, *p* = 0.016), depression (r = −0.079, *p* < 0.001), and stress (r = −0.105, *p* < 0.001) symptoms. Conversely, a significant positive correlation with subjective well-being (r = 0.049, *p* = 0.02) was estimated. These results demonstrated a significant association of a positive psychological profile during the lockdown with the tendency to adopt healthier and more sustainable food consumption behaviors.

Table 5 shows the results that emerged from the multiple linear regression analysis. The simultaneous impact of the socio-demographic, household and clinical characteristics and the psychological variables on the HST index scores was explored. Significant associations were found with the BMI (b = −0.013, SE = 0.006, *p* = 0.022) and the stress symptoms (b = −0.033, SE = 0.009, *p* < 0.001), meaning that increased weight and more severe stress symptomatology significantly contributed to worsen healthy-sustainable dietary behaviors. A significant explained variance emerged from the model (*R*^2^ = 0.021, F_(11,2050)_ = 4.023, *p* < 0.001), and a small effect size was estimated (f^2^ = 0.02). A post-hoc power analysis was then performed to calculate the achieved statistical power of the regression model. The model showed satisfactory results (1-β = 0.99).

## 4. Discussion

The present research had a twofold purpose. Firstly, it aimed to describe the effects of the earlier phase of COVID-19 pandemic (March–May 2020) on food consumption behaviors in a convenience sample of Italian residents. This observation specifically regarded the dietary changes in terms of healthiness and sustainability. For this purpose, the EATLancet Commission’s guidelines [31] for a planetary healthy-sustainable diet were followed, thereby investigating participants’ dietary habits and their change based on sustainable (i.e., vegetable-based dishes, legumes, whole grain cereals, nuts and seeds, fresh fruit) and unsustainable (i.e., carb-based dishes, meat-based dishes, dairy products, sweets and desserts, alcoholic and sugary beverages) foods consumption. Secondly, the present survey aimed to explore the associations of the dietary changes observed with the psychological responses to the pandemic. These latter were assessed in relation to the lockdown period when participants were housebound and could go outside only for primary necessity activities (i.e., going to work, reaching hospitals, and grocery shopping). The evaluation consisted in the measurement of subjective well-being, depression, anxiety and stress symptomatology.

Regarding the first aim, an aggregate indicator of healthy-sustainable transition (HST index) of dietary behaviors was calculated. This made it possible to investigate the changes in food consumption behaviors during the lockdown by comparing them with those adopted before the pandemic. The results of the HST index revealed that most of the study sample perceived no effects of pandemic-related restrictions on their dietary habits, meaning that the consumption of sustainable and unsustainable foods has not changed in terms of frequency alongside the COVID-19 outbreak. Notably, a considerable number of participants showed a transition, however, reflecting a diffused transformation in nutrition conducts. Of these, some started adopting healthier and more sustainable dietary behaviors, while others reduced healthy-sustainable food choices. This finding is in line with a prior study that has so far reported mixed evidence on this topic, especially when referring to non-clinical populations. Accordingly, while some prior studies showed improvements in the adherence to healthy diets [38,39], some others reported a more frequent food intake, along with an increase in ultra-processed food and alcohol consumption, ultimately leading to higher caloric intake [22,40,41,42,43]. In contrast to this, further works evidenced patterns of stability on eating behaviors in spite of a pandemic outbreak [44,45,46]. So far, the heterogeneity observed in the trajectories of eating behaviors has been partly explained by the role of some socio-demographic and clinical characteristics, such as age, gender, working status, and BMI. In the present study, substantial effects of these factors were observed with men, younger participants, those working from home, and those with a lower BMI displaying a significantly more positive transitions towards healthy-sustainable food consumption behaviors than the other subgroups. The effect of gender, for example, provided an informative result as, in prior works, it was evidenced as a variable not significantly correlating with specific eating behaviors [3]. It remains, however, a finding to take cautiously, since our study sample predominantly consisted of women. Similarly, the result on age is in contrast with prior literature reporting an association between young people and lower adherence to healthy diets mainly related to the tendency to increase food intake, the preference for snacks, and the decrease in fruit and vegetable consumption [47,48,49,50]. Regarding the role of working condition, our finding, on the one hand, is in contrast with prior research explaining that spending more time at home contributed to increasing self-made foods consumption that, in some cases, appeared to be correlated to the use of food delivery services [43,51]. On the other hand, staying at home has represented in some other cases an opportunity for re-discovering cooking habits with a positive impact on the attention for diet quality, as suggested in a prior work [13]. Lastly, the evidence on the effect of the BMI corroborates what was found in previous studies suggesting that people with obesity showed worse eating behaviors with an increase in the frequency and amount of unhealthy food products [52,53]. Overall, based on these mixed findings, what is the role of socio-demographic and clinical variables and what are their associations with eating behaviors changes during pandemic era still remain open questions. Future studies should take into account and address this heterogeneity in order to draw more generalizable conclusions on the impact of such factors.

Concerning the second aim, association analyses were conducted to explore the psychological factors underlying the changes observed on healthy-sustainable food consumption behaviors. Diverse studies have so far emphasized the importance of also considering mental health and psychological factors in the study of COVID-19 pandemic-related health behavior changes [42,54]. The investigation on the role of psychological factors has been driven by the interest to understand whether and to what extent an unprecedented event like the COVID-19 pandemic affected people’s everyday life, including their daily routines in terms of healthy behaviors [55]. Plenty of studies explored the role of different psychological constructs in relation to the pandemic, both in clinical [56] and non-clinical [57] populations, ultimately concluding that mental health represents a crucial issue to be addressed when aiming to preserve healthy lifestyles and behaviors. The present study specifically explored the mental health factors associated with eating behavior changes in terms of healthiness and sustainability by including psychological constructs like subjective well-being and depression, anxiety and stress symptomatology derived from COVID-19-related restrictions.

Correlational analyses revealed significant associations of all constructs with the HST index, suggesting that a more positive psychological profile contributed not only to adopting healthier food consumption behaviors, but also to making more sustainable food choices. Prior works support these findings by underlining the effects of emotional aspects on food habits and choices [14,15,17,58]. Accordingly, it is acknowledged how experiencing negative emotions may lead to overeating. For example, contextually to the COVID-19 pandemic era, self-isolation during the lockdown may have triggered the tendency to look for reward and gratification through food consumption [59]. Similarly, in order to contrast boredom feelings deriving from staying home for an extended period may have contributed to overeating to escape monotony [60,61]. Notably, perceived stress has been evidenced as one of the most common symptoms alongside the pandemic outbreak. Especially in the earlier phases of pandemic when most of COVID-19 issues, including its short- and long-term effects, were still unknown, stress may have increased because of the fear of contracting the virus and worrying about relatives. Again, loneliness or economic uncertainty may have had an effect, ultimately causing stressful responses that, in turn, have affected lifestyle behavior regulation [23,62].

The negative association between higher stress symptomatology and healthy food consumption behaviors was confirmed in our study as well. Controlling for all the socio-demographic and clinical characteristics of the study sample, the multiple linear regression analysis performed has outlined a significant impact of stress scores on the HST index, meaning that higher stress contributed to worsen healthy-sustainable food choices. In the same statistical model, another significant effect emerged from the association with BMI. As previously mentioned, higher body weight was suggested to be associated with worse eating behaviors along with the increase in the amount and frequency of unhealthy foods. The significant effect we have found in our analysis corroborates this association, additionally providing further insights into the simultaneous effects of the psychological factors. Consistently, prior works recognized the association between stress, eating behaviors, and body composition, specifically suggesting that stressful events may trigger emotional eating and the higher intake of hedonic food and snacks, ultimately leading to weight gain [63]. Regarding the role of subjective well-being, and depression and anxiety symptoms, although significant correlations with the HST index were found, they did not result as independent predictors. Firstly, it must be noted that the effects estimated from the correlational analyses were particularly weak. Secondly, it should be underlined that, according to prior research [64,65,66], anxiety and depression symptoms were evidenced as factors predisposing to higher COVID-19 pandemic psychological impact mainly in vulnerable populations due to their belief of being at higher exposure to experience worse prognoses through the COVID-19 infection course. The present survey was conducted on a sample of healthy participants, leading us to infer that the effects of depression and anxiety symptoms have been mitigated by the typology of the population involved. Similarly, subjective well-being was not significantly associated with the HST index. This result is not surprising if we consider that the study sample mainly declared that no effects of COVID-19 crisis on socio-economic condition occurred. This may support, therefore, the absence of a significant effect on healthy-sustainable dietary behavior changes. Overall, given the aforementioned main prevalence of stress symptoms in the general population during the earlier phases of the pandemic, we infer that stress may have had a larger effect on healthy-sustainable food consumption behaviors than the other psychological constructs, making it a predominant predisposing factor. This result becomes more interesting if we consider that the number of cases reporting stress symptomatology in this study was relatively low. Despite the prevalence observed, stress appeared as a significant factor affecting healthy-sustainable choices. 

The study has some limitations. First, despite the fact that the evaluation of healthy-sustainable dietary behaviors was purposely designed to outline the changes that occurred during pandemic, it must be acknowledged that a cross-sectional design was adopted, therefore making it impossible to accurately establish temporal relationships with the psychological factors included. Accordingly, similar studies adopting a longitudinal approach are needed. Moreover, data collection relied on self-reports from participants that may have led to methodological and inferential limitations (e.g., social desirability bias). However, it is noteworthy that the self-report method has revealed to be suitable to provide steps in understanding a phenomenon [67], thanks to significant advantages (e.g., high practicality of use, clinical and research applicability, and good cost-effectiveness). Lastly, a convenience sample was involved. Although non-probability sampling may have represented a limit, convenience samples allow for the investigation in the early stages of research a novel or growing phenomenon [68]. For the present work, convenience sampling made it possible to explore a new topic such as the relationships between psychological factors and healthy-sustainable food consumption transitions immediately after the COVID-19 outbreak. 

Despite the limitations, the present study provided informative insights into the psychological correlates of dietary behavior change not only in terms of healthiness, but most importantly in terms of sustainability. This made it possible to identify crucial areas of future institutional interventions aiming to ensure favorable conditions for healthier eating behaviors and sustainable food consumption choices. Furthermore, a large sample size was included. This allowed for a more precise estimate of the effects observed and to draw more generalizable conclusions. Also, including a convenience sample helped to optimally interpret emerging associations that, in future studies, will be more rigorously explored by the inclusion of probability samples.

## 5. Conclusions

The present study showed diffused transformation in healthy-sustainable dietary habits during the earlier phases of the COVID-19 pandemic. A large segment of the study sample reported no relevant dietary changes during the lockdown. Of the remaining participants, while some adopted healthier and more sustainable eating behaviors, some others declared that they had worsened. Informative evidence on the psychological correlates of such transformation emerged. Subjective well-being, and depression, anxiety and stress symptoms were found to be significantly correlated to healthy-sustainable food consumption behavior changes. Specifically, stress symptomatology was estimated as a predominant factor significantly predisposing individuals to worse healthy-sustainable eating behavior profiles. These associations shed light on crucial areas of institutional intervention (i.e., age, working condition, BMI, and the psychological status) to target in the future with the purpose of ensuring more advantageous conditions for healthier and more sustainable food choices. Following this line, future steps may, for example, consist of the development of effective communication strategies particularly intended for specific segments of the general population. This would allow to implement a tailored approach that is able to promote the adherence to health and sustainability claims. Contextually, the introduction of innovative technological tools (e.g., smartphone apps) may represent a valuable strategy in order to profile individuals and facilitate positive transitions. As suggested by prior works, web-based tailored health communication interventions have already shown their usefulness [69]. Future studies and practice should investigate the effectiveness of such strategies in the promotion of health-related choices contributing to responding concurrently to the issues related to sustainability. 

## Figures and Tables

**Table 1:** Socio-demographic, household, and clinical characteristics of the study sample (*n* = 2272).

Variables	*n*(%)
Generational cohort	
*Gen Z (18–24)*	418(18.4)
*Young Millennials (25–29)*	348(15.3)
*Adult Millennials (30–40)*	570(25.1)
*Gen X (41–55)*	604(26.6)
*Baby Boomers (56–76)*	321(14.1)
*Elderly (over 77)*	11(0.5)
Gender	
*Male*	605(26.7)
*Female*	1664(73.3)
Place of Residency	
*Lombardy region*	1543(67.9)
*Other regions in Northern Italy*	341(15.0)
*Central Italy*	137(6.1)
*Southern Italy*	251(11.0)
Educational level	
*Up to middle school*	68(3.0)
*High School*	668(29.4)
*Graduate*	972(42.8)
*Post-graduate*	564(24.8)
Effect of COVID-19 crisis on household economic condition	
*Strongly detrimental*	213(9.4)
*Slightly detrimental*	660(29.0)
*No effect*	1234(54.3)
*Slightly beneficial*	154(6.8)
*Strongly beneficial*	11(0.5)
Working condition under lockdown	
*From home (always or most days)*	1355(59.6)
*Not working (on leave, unemployed etc.)*	264(11.6)
*Essential sector (working as usual)*	183(8.1)
*Other (students, retired, or unspecified)*	470(20.7)
Household composition	
*Single*	262(11.7)
*Couple*	731(32.7)
*3 people*	539(24.2)
*4–5 people*	654(29.3)
*6 people or more*	48(2.1)
Body Mass Index (BMI)	
*Underweight*	103(4.5)
*Normal weight*	1382(60.9)
*Overweight*	445(19.6)
*Obese*	341(15.0)

**Table 2:** Distribution of dietary habits of the study sample within sustainable and unsustainable food groups.

Sustainable Foods	*n*(%)	Unsustainable Foods	*n*(%)
Vegetable-based dishes		Carb-based dishes	
*Lower consumption*	229(10.1)	*Lower consumption*	183(8.1)
*No change*	1279(56.3)	*No change*	1380(60.7)
*Higher consumption*	739(32.5)	*Higher consumption*	691(30.4)
*Never consumed*	25(1.1)	*Never consumed*	18(0.8)
Legumes		Meat-based dishes	
*Lower consumption*	256(11.3)	*Lower consumption*	354(15.6)
*No change*	1291(56.8)	*No change*	1397(61.5)
*Higher consumption*	526(23.2)	*Higher consumption*	334(14.7)
*Never consumed*	199(8.8)	*Never consumed*	187(8.2)
Whole grain cereals		Dairy products	
*Lower consumption*	261(11.5)	*Lower consumption*	243(10.7)
*No change*	1360(59.9)	*No change*	1395(61.4)
*Higher consumption*	317(14.0)	*Higher consumption*	498(21.9)
*Never consumed*	3341(4.7)	*Never consumed*	136(6.0)
Nuts and oil seeds		Sweets and desserts	
*Lower consumption*	345(15.2)	*Lower consumption*	327(14.4)
*No change*	1106(48.7)	*No change*	784(34.5)
*Higher consumption*	342(15.1)	*Higher consumption*	1067(47.0)
*Never consumed*	479(21.1)	*Never consumed*	94(4.1)
Fresh fruits		Alcoholic/sugary beverages	
*Lower consumption*	235(10.3)	*Lower consumption*	546(24.0)/278(12.2)
*No change*	1346(59.2)	*No change*	681(30.0)/580(25.5)
*Higher consumption*	625(27.5)	*Higher consumption*	443(19.5)/220(9.7)
*Never consumed*	66(2.9)	*Never consumed*	602(26.5)/1194(52.6)

**Table 3:** Between-group comparisons on the HST index means scores and the psychological variables.

Variable	*n*(%)	Mean(SD)	*F*	*p*
Subjective well-being				
*≤4*	255(11.3)	−0.06(1.0)	2.400	0.048
*5*	409(18.1)	−0.01(0.9)		
*6*	593(26.2)	−0.08(0.9)		
*7*	715(31.6)	0.07(1.0)		
*≥8*	291(12.6)	0.08(0.9)		
Anxiety				
*Normal*	1584(75.0)	0.03(0.9)	2.129	0.075
*Mild*	260(12.3)	−0.08(1.0)		
*Moderate*	115(5.4)	−0.06(1.1)		
*Severe*	74(3.5)	−0.24(1.0)		
*Extremely Severe*	78(3.7)	−0.01(1.2)		
Depression				
*Normal*	1218(57.7)	0.06(0.9) *°	3.355	0.010
*Mild*	342(16.2)	−0.05(1.0)		
*Moderate*	344(16.3)	−0.01(1.0)		
*Severe*	127(6.0)	−0.18(1.0) *		
*Extremely Severe*	80(3.8)	−0.23(1.2) °		
Stress				
*Normal*	1521(76.4)	0.04(1.0) *	2.598	0.035
*Mild*	228(11.4)	0.07(1.1)		
*Moderate*	85(4.3)	−0.16(0.9)		
*Severe*	132(6.6)	−0.19(1.0) *		
*Extremely Severe*	25(1.3)	0.01(1.4)		

Notes. Means scores and Standard Deviations (SD) of the HST index are reported. A one-way ANOVA test was conducted with the psychological variables as independent factors and the HST as dependent factor. F, Fisher’s coefficient. * and °, Between-groups means differences are significant (*p* < 0.05) at multi comparisons LSD post-hoc test procedures.

**Table 4:** Pearson’s correlation coefficients of HST index scores and the psychological variables.

	1	2	3	4	5
1. HST index	-				
2. Anxiety	−0.053 *	-			
3. Depression	−0.079 **	0.633 **	-		
4. Stress	−0.105 **	0.700 **	0.714 **	-	
5. Subjective well-being	0.049 *	−0.144 **	−0.168 **	−0.128 **	-

Notes. * *p* < 0.05. ** *p* < 0.001.

**Table 5:** Multiple linear regression analyses with HST index as dependent variable.

Variables	B	SE	*t*	95% CI		*p*
Age	−0.003	0.001	−1.834	−0.006	2.260	0.067
Gender (ref: Male)	−0.059	0.052	−1.128	−0.162	0.043	0.259
Educational level (ref: Post-graduate)						
*Up to middle school*	0.108	0.143	0.754	−0.173	0.388	0.451
*High school*	0.043	0.064	0.674	−0.083	0.169	0.501
*Graduate*	0.004	0.057	0.075	−0.107	0.116	0.940
Household economic condition (ref: Strongly beneficial)						
*Strongly detrimental*	−0.009	0.361	−0.027	−0.718	0.698	0.978
*Slightly detrimental*	−0.120	0.354	−0.339	−0.815	0.575	0.723
*No effect*	−0.068	0.353	−0.192	−0.760	0.625	0.848
*Slightly beneficial*	−0.083	0.362	−0.228	−0.791	0.627	0.819
Working condition under lockdown (ref: From home)						
*Not working (on leave, unemployed etc.)*	−0.136	0.076	−1.799	−0.284	0.012	0.072
*Essential sector (working as usual)*	−0.257	0.083	−3.108	−0.419	−0.095	0.002
*Other (students, retired, or unspecified)*	−0.055	0.060	−0.917	−0.172	0.062	0.359
Household composition (ref: Single)						
*Couple*	−0.039	0.075	−0.522	−0.186	0.108	0.602
*3 people*	−0.061	0.079	−0.763	−0.216	0.095	0.446
*4–5 people*	−0.110	0.079	−1.390	−0.264	0.045	0.165
*6 people or more*	−0.012	0.165	−0.072	−0.335	0.312	0.943
BMI	−0.013	0.006	−2.288	−0.024	−0.002	0.022
Anxiety	0.019	0.011	1.645	−0.004	0.041	0.100
Depression	−0.006	0.008	−0.714	−0.023	0.011	0.475
Stress	−0.033	0.009	−3.682	−0.051	−0.016	<0.001
Subjective well-being	0.028	0.017	1.612	−0.006	0.006	0.107

Notes. B, unstandardized regression coefficient. SE, Standard Error. *t*, Student’s *t* coefficient. CI, Coefficient Interval. All predictors were defined as continuous variables, except for gender (dichotomous variable), and educational level, household economic condition, working condition under lockdown, and household composition (multi-levels categorical variables).

## Data Availability

Data used in this report may be available upon requests made to the corresponding author.

## Supplementary Materials

The following supporting information can be downloaded at: https://www.mdpi.com/article/10.3390/foods11131944/s1. Table S1: Results of the CatPCA on the 5 items of the sustainable food groups; Table S2: CatPCA component loadings for the five items (sustainable food group) for the first component; Table S3: Results of the CatPCA on the 6 items of the non-sustainable food group; Table S4: CatPCA component loadings for the six items (non-sustainable food group) respect the first component; Table S5: Results of the PCA on the CCSF and CCNSF; Table S6: Between-group comparisons on the HST index means scores and the socio-demographic, household, and clinical variables.

## References

[1] Remuzzi A., Remuzzi G. (2020). COVID-19 and Italy: What Next?. Lancet.

[2] Mattioli A.V., Ballerini Puviani M., Nasi M., Farinetti A. (2020). COVID-19 pandemic: The effects of quarantine on cardiovascular risk. Eur. J. Clin. Nutr..

[3] González-Monroy C., Gómez-Gómez I., Olarte-Sánchez C.M., Motrico E. (2021). Eating Behaviour Changes during the COVID-19 Pandemic: A Systematic Review of Longitudinal Studies. Int. J. Environ. Res. Public Health.

[4] Mignogna C., Costanzo S., Ghulam A., Cerletti C., Donati M.B., de Gaetano G., Iacoviello L., Bonaccio M. (2021). Impact of Nationwide Lockdowns Resulting from the First Wave of the COVID-19 Pandemic on Food Intake, Eating Behaviors, and Diet Quality: A Systematic Review. Adv. Nutr..

[5] Galimberti A., Cena H., Campone L., Ferri E., Dell’Agli M., Sangiovanni E., Belingheri M., Riva M.A., Casiraghi M., Labra M. (2020). Rethinking Urban and Food Policies to Improve Citizens Safety After COVID-19 Pandemic. Front. Nutr..

[6] Laviano A., Koverech A., Zanetti M. (2020). Nutrition support in the time of SARS-CoV-2 (COVID-19). Nutrition.

[7] Rebelos E., Moriconi D., Virdis A., Taddei S., Foschi D., Nannipieri M. (2020). Letter to the Editor: Importance of metabolic health in the era of COVID-19. Metabolism.

[8] Muscogiuri G., Barrea L., Savastano S., Colao A. (2020). Nutritional recommendations for COVID-19 quarantine. Eur. J. Clin. Nutr..

[9] Naja F., Hamadeh R. (2020). Nutrition amid the COVID-19 pandemic: A multilevel framework for action. Eur. J. Clin. Nutr..

[10] Cena H., Chieppa M. (2020). Coronavirus Disease (COVID-19–SARS-CoV-2) and Nutrition: Is Infection in Italy Suggesting a Connection?. Front. Immunol..

[11] FAO., WHO (2019). Sustainable Healthy Diets—Guiding Principles.

[12] Meybeck A., Gitz V. (2017). Sustainable diets within sustainable food systems. Proc. Nutr. Soc..

[13] Matacena R., Zenga M., D’Addario M., Mari S., Labra M. (2021). COVID-19 as an Opportunity for a Healthy-Sustainable Food Transition. An Analysis of Dietary Transformations during the First Italian Lockdown. Sustainability.

[14] Slurink I., Smaardijk V.R., Kop W.J., Kupper N., Mols F., Schoormans D., Soedamah-Muthu S.S. (2022). Changes in Perceived Stress and Lifestyle Behaviors in Response to the COVID-19 Pandemic in The Netherlands: An Online Longitudinal Survey Study. Int. J. Environ. Res. Public Health.

[15] de Souza Cunha C., Haikal D.S., Silva R., de Pinho L., das Graças Pena G., Bicalho A.H., de Souza Costa Sobrinho P., Nobre L.N. (2022). Association between lifestyle and emotional aspects of food consumption during the COVID-19 pandemic. Nutr. Metab. Cardiovasc. Dis..

[16] Caroppo E., Mazza M., Sannella A., Marano G., Avallone C., Claro A.E., Janiri D., Moccia L., Janiri L., Sani G. (2021). Will Nothing Be the Same Again?: Changes in Lifestyle during COVID-19 Pandemic and Consequences on Mental Health. Int. J. Environ. Res. Public Health.

[17] López-Moreno M., López M.T.I., Miguel M., Garcés-Rimón M. (2020). Physical and Psychological Effects Related to Food Habits and Lifestyle Changes Derived from COVID-19 Home Confinement in the Spanish Population. Nutrients.

[18] Lima C.K., de Medeiros Carvalho P.M., Lima I.D., de Oliveira Nunes J.V., Saraiva J.S., de Souza R.I., da Silva C.G., Neto M.L. (2020). The Emotional Impact Of Coronavirus 2019-Ncov (New Coronavirus Disease). Psychiatry Res..

[19] Sánchez-Sánchez E., Ramírez-Vargas G., Avellaneda-López Y., Orellana-Pecino J.I., García-Marín E., Díaz-Jimenez J. (2020). Eating Habits and Physical Activity of the Spanish Population during the COVID-19 Pandemic Period. Nutrients.

[20] Di Renzo L., Gualtieri P., Pivari F., Soldati L., Attinà A., Cinelli G., Leggeri C., Caparello G., Barrea L., Scerbo F. (2020). Eating habits and lifestyle changes during COVID-19 lockdown: An Italian survey. J. Transl. Med..

[21] Ben Hassen T., El Bilali H., Allahyari M.S. (2020). Impact of COVID-19 on Food Behavior and Consumption in Qatar. Sustainability.

[22] Deschasaux-Tanguy M., Druesne-Pecollo N., Esseddik Y., de Edelenyi F.S., Allès B., Andreeva V.A., Baudry J., Charreire H., Deschamps V., Egnell M. (2021). Diet and physical activity during the coronavirus disease 2019 (COVID-19) lockdown (March-May 2020): Results from the French NutriNet-Santé cohort study. Am. J. Clin. Nutr..

[23] Chopra S., Ranjan P., Singh V., Kumar S., Arora M., Hasan M.S., Kasiraj R., Suryansh, Kaur D., Vikram N.K. (2020). Impact of COVID-19 on lifestyle-related behaviours- a cross-sectional audit of responses from nine hundred and ninety-five participants from India. Diabetes Metab. Syndr..

[24] Gazzetta Ufficiale. https://www.gazzettaufficiale.it/eli/id/2020/03/09/20A01558/sg.

[25] Adler N.E., Epel E.S., Castellazzo G., Ickovics J.R. (2000). Relationship of subjective and objective social status with psychological and physiological functioning: Preliminary data in healthy, White women. Health Psychol..

[26] Präg P., Mills M.C., Wittek R. (2016). Subjective socioeconomic status and health in cross-national comparison. Soc. Sci. Med..

[27] Navarro-Carrillo G., Alonso-Ferres M., Moya M., Valor-Segura I. (2020). Socioeconomic Status and Psychological Well-Being: Revisiting the Role of Subjective Socioeconomic Status. Front. Psychol..

[28] Bottesi G., Ghisi M., Altoè G., Conforti E., Melli G., Sica C. (2015). The Italian version of the Depression Anxiety Stress Scales-21: Factor structure and psychometric properties on community and clinical samples. Compr. Psychiatry.

[29] Shah S.M.A., Mohammad D., Qureshi M.F.H., Abbas M.Z., Aleem S. (2021). Prevalence, Psychological Responses and Associated Correlates of Depression, Anxiety and Stress in a Global Population, During the Coronavirus Disease (COVID-19) Pandemic. Community Ment. Health J..

[30] Gliem J.A., Gliem R.R. (2003). Calculating, Interpreting, and Reporting Cronbach’s Alpha Reliability Coefficient for Likert-type Scales. Midwest Research-to-Practice Conference in Adult, Continuing, and Community Education.

[31] Willett W., Rockström J., Loken B., Springmann M., Lang T., Vermeulen S., Garnett T., Tilman D., DeClerck F., Wood A. (2019). Food in the Anthropocene: The EAT–Lancet Commission on healthy diets from sustainable food systems. Lancet.

[32] Linting M., Van Der Kooij A. (2012). Nonlinear Principal Components Analysis With CATPCA: A Tutorial. J. Personal. Assess..

[33] Linting M., Meulman J.J., Groenen P.J.F., Van Der Koojj A.J. (2007). Nonlinear principal components analysis: Introduction and application. Psychol. Methods.

[34] Meulman J.J., Van der Kooij A.J., Heiser W.J., Kaplan D. (2004). Principal components analysis with nonlinear optimal scaling transfor-mations for ordinal and nominal data. The Sage Handbook of Quantitative Methodology for the Social Sciences.

[35] Cronbach L.J. (1951). Coefficient alpha and the internal structure of tests. Psychometrika.

[36] Heiser W.J., Meulman J.J., Greenacre M., Blasius J. (1992). Homogeneity analysis: Exploring the distribution of variables and their nonlinear relationships. Correspondence Analysis in the Social Sciences: Recent Developments and Applications.

[37] World Health Organization A Healthy Lifestyle—WHO Recommendations. https://www.who.int/europe/news-room/fact-sheets/item/a-healthy-lifestyle.

[38] Zhang J., Zhao A., Ke Y., Huo S., Ma Y., Zhang Y., Ren Z., Li Z., Liu K. (2020). Dietary behaviors in the post-lockdown period and its effects on dietary diversity: The second stage of a nutrition survey in a longitudinal chinese study in the COVID-19 era. Nutrients.

[39] Lamarche B., Brassard D., Lapointe A., Laramée C., Kearney M., Côté M., Bélanger-Gravel A., Desroches S., Lemieux S., Plante C. (2021). Changes in diet quality and food security among adults during the COVID-19-related early lockdown: Results from NutriQuébec. Am. J. Clin. Nutr..

[40] Maffoni S., Brazzo S., De Giuseppe R., Biino G., Vietti I., Pallavicini C., Cena H. (2021). Lifestyle changes and body mass index during COVID-19 pandemic lockdown: An Italian online-survey. Nutrients.

[41] Czenczek-Lewandowska E., Wyszyńska J., Leszczak J., Baran J., Weres A., Mazur A., Lewandowski B. (2021). Health behaviours of young adults during the outbreak of the COVID-19 pandemic—A longitudinal study. BMC Public Health.

[42] Herle M., Smith A.D., Bu F., Steptoe A., Fancourt D. (2021). Trajectories of eating behavior during COVID-19 lockdown: Longitudinal analyses of 22,374 adults. Clin. Nutr. ESPEN.

[43] Sato K., Kobayashi S., Yamaguchi M., Sakata R., Sasaki Y., Murayama C., Kondo N. (2021). Working from home and dietary changes during the COVID-19 pandemic: A longitudinal study of health app (CALO mama) users. Appetite.

[44] Munasinghe S., Sperandei S., Freebairn L., Conroy E., Jani H., Marjanovic S., Page A. (2020). The Impact of Physical Distancing Policies During the COVID-19 Pandemic on Health and Well-Being Among Australian Adolescents. J. Adolesc. Health.

[45] Curtis R.G., Olds T., Ferguson T., Fraysse F., Dumuid D., Esterman A., Hendrie G.A., Brown W.J., Lagiseti R., Maher C.A. (2021). Changes in diet, activity, weight, and wellbeing of parents during COVID-19 lockdown. PLoS ONE.

[46] Barone Gibbs B., Kline C.E., Huber K.A., Paley J.L., Perera S. (2021). COVID-19 shelter-at-home and work, lifestyle and well-being in desk workers. Occup. Med..

[47] Imaz-Aramburu I., Fraile-Bermúdez A.B., Martín-Gamboa B.S., Cepeda-Miguel S., Doncel-García B., Fernandez-Atutxa A., Irazusta A., Zarrazquin I. (2021). Influence of the COVID-19 pandemic on the lifestyles of health sciences university students in spain: A longitudinal study. Nutrients.

[48] Yu B., Zhang D., Yu W., Luo M., Yang S., Jia P. (2021). Impacts of lockdown on dietary patterns among youths in China: The COVID-19 Impact on Lifestyle Change Survey. Public Health Nutr..

[49] Jia P., Liu L., Xie X., Yuan C., Chen H., Guo B., Zhou J., Yang S. (2021). Changes in dietary patterns among youths in China during COVID-19 epidemic: The COVID-19 impact on lifestyle change survey (COINLICS). Appetite.

[50] Dun Y., Ripley-Gonzalez J.W., Zhou N., You B., Li Q., Li H., Zhang W., Thomas R.J., Olson T.P., Liu J. (2021). Weight gain in Chinese youth during a 4-month COVID-19 lockdown: A retrospective observational study. BMJ Open.

[51] Skotnicka M., Karwowska K., Kłobukowski F., Wasilewska E., Małgorzewicz S. (2021). Dietary Habits before and during the COVID-19 Epidemic inSelected European Countries. Nutrients.

[52] Pellegrini M., Ponzo V., Rosato R., Scumaci E., Goitre I., Benso A., Belcastro S., Crespi C., De Michieli F., Ghigo E. (2020). Changes in weight and nutritional habits in adults with obesity during the “lockdown” period caused by the COVID-19 virus emergency. Nutrients.

[53] Li X., Li J., Qing P., Hu W. (2021). COVID-19 and the Change in Lifestyle: Bodyweight, Time Allocation, and Food Choices. Int. J. Environ. Res. Public Health.

[54] Naughton F., Ward E., Khondoker M., Belderson P., Marie Minihane A., Dainty J., Hanson S., Holland R., Brown T., Notley C. (2021). Health behaviour change during the UK COVID-19 lockdown: Findings from the first wave of the C-19 health behaviour and well-being daily tracker study. Br. J. Health Psychol..

[55] Bentlage E., Ammar A., How D., Ahmed M., Trabelsi K., Chtourou H., Brach M. (2020). Practical recommendations for maintaining active lifestyle during the COVID-19 pandemic: A systematic literature review. Int. J. Environ. Res. Public Health.

[56] Palmer K., Monaco A., Kivipelto M., Onder G., Maggi S., Michel J.P., Prieto R., Sykara G., Donde S. (2020). The potential long-term impact of the COVID-19 outbreak on patients with non-communicable diseases in Europe: Consequences for healthy ageing. Aging Clin. Exp. Res..

[57] Xiong J., Lipsitz O., Nasri F., Lui L., Gill H., Phan L., Chen-Li D., Iacobucci M., Ho R., Majeed A. (2020). Impact of COVID-19 pandemic on mental health in the general population: A systematic review. J. Affect. Disord..

[58] Evers C., Dingemans A., Junghans A.F., Boevé A. (2018). Feeling bad or feeling good, does emotion affect your consumption of food? A meta-analysis of the experimental evidence. Neurosci. Biobehav. Rev..

[59] Alamri E.S. (2021). Effects of COVID-19 home confinement on eating behavior: A review. J. Public Health Res..

[60] Havermans R.C., Vancleef L., Kalamatianos A., Nederkoorn C. (2015). Eating and inflicting pain out of boredom. Appetite.

[61] Crockett A.C., Myhre S.K., Rokke P.D. (2015). Boredom proneness and emotion regulation predict emotional eating. J. Health Psychol..

[62] Hall P.A., Sheeran P., Fong G.T., Cheah C.S.L., Oremus M., Liu-Ambrose T., Sakib M.N., Butt Z.A., Ayaz H., Jandu N. (2021). Biobehavioral Aspects of the COVID-19 Pandemic: A Review. Psychosom. Med..

[63] Hootman K.C., Guertin K., Cassano P.A. (2018). Stress and psychological constructs related to eating behavior are associated with anthropometry and body composition in young adults. Appetite.

[64] Sensoy B., Gunes A., Ari S. (2021). Anxiety and depression levels in COVID-19 disease and their relation to hypertension. Clin. Exp. Hypertens.

[65] D’Addario M., Zanatta F., Adorni R., Greco A., Fattirolli F., Franzelli C., Giannattasio C., Steca P. (2021). Depression symptoms as longitudinal predictors of the psychological impact of COVID-19 pandemic in hypertensive patients. Sci. Rep..

[66] Tian W., Jiang W., Yao J., Nicholson C.J., Li R.H., Sigurslid H.H., Wooster L., Rotter J.I., Guo X., Malhotra R. (2020). Predictors of mortality in hospitalized COVID-19 patients: A systematic review and meta-analysis. J. Med. Virol..

[67] Paulhus D.L., Vazire S., Robins R.W., Fraley R.C., Krueger R. (2007). The self-report method. Handbook of Research Methods in Personality Psychology.

[68] Fricker R.D., Schonlau M. (2002). Advantages and Disadvantages of Internet Research Surveys: Evidence from the Literature. Field Methods.

[69] Lee M., Lee H., Kim Y., Kim J., Cho M., Jang J., Jang H. (2018). Mobile app-based health promotion programs: A systematic review of the literature. Int. J. Environ. Res. Public Health.

