# Supplementary material for: The Role of Selected Psychological Factors in Healthy-Sustainable Food Consumption Behaviors during the COVID-19 Pandemic
Source: Foods. 2022 Jun 29;11(13):1944. doi: 10.3390/foods11131944 (PMC9265338; doi:10.3390/foods11131944)
Supplement: Supplementary file 1 [file foods-11-01944-s001.zip › foods-1766701-supplementary.pdf]

Supplementary Materials

Table S1. Results of the CatPCA on the 5 items of the sustainable food groups.

Dimension	Cronbach's Alpha	Eigenvalues	% Variance
1	0.587	1.884	37.689
2	-0.077	0.942	18.834
3	-0.162	0.886	17.71
4	-0.513	0.709	14.178
5	-0.907	0.579	11.589
Total	1	5	100

Table S2. CatPCA component loadings for the five items (sustainable food group) for the first component.

Change on food consumption	Component loading
Vegetables-based dishes	0.710
Legumes	0.696
Whole grain cereals	0.619
Fresh fruits	0.585
Nuts and oil seeds	0.425

Table S3. Results of the CatPCA on the 6 items of the non-sustainable food group.

Dimension	Cronbach's Alpha	Eigenvalues	% Variance
1	0.510	1.739	28.990
2	-0.006	0.995	16.584
3	-0.053	0.958	15.964
4	-0.249	0.828	13.804
5	-0.346	0.776	12.936
6	-0.506	0.703	11.722
Total	1	6	100

Table S4. CatPCA component loadings for the six items (non-sustainable food group) respect the first component.

Change on food consumption	Component loading
Carb-based dishes	0.628
Meat-based dishes	0.378
Dairy products	0.576
Sweets and desserts	0.664
Sugary beverage	0.515

Table S5. Results of the PCA on the CCSF and CCNSF

Dimension	Eigenvalues	% Variance
1	1.188	59.402
2	0.812	40.598
Total	2	100

Table S6. Between-group comparisons on the HST index means scores and the socio-demographic, household, and clinical variables.

**Variable**	**Mean(SD)**	**F**	***p***
Generational cohort		2.751	0.017
Gen Z (18-24)	0.12(1.0)*°		
Young Millennials (25-29)	0.03(1.0)		
Adult Millennials (30-40)	-0.07(1.0)*		
Gen X (41-55)	-0.07(1.0)°		
Baby Boomers (56-76)	0.05(0.9)		
Elderly (over 77)	-0.05(0.6)		
Educational level		0.105	0.957
Up to middle school	0.03(0.9)		
High School	-0.01(1.0)		
Graduate	-0.01(1.0)		
Post-graduate	0.02(0.9)		
Household economic condition		1.068	0.371
Strongly detrimental	0.01(1.1)		
Slightly detrimental	-0.07(0.9)		
No effect	0.03(1.0)		
Slightly beneficial	0.05(1.0)		
Strongly beneficial	0.04(1.3)		
Working condition		3.159	0.024
From home (always or most days)	0.04(1.0)*		
Not working (on leave, unemployed etc.)	-0.04(1.1)		
Essential sector (working as usual)	-0.20(0.9)*		
Other (students, retired, or unspecified)	-0.01(0.9)		
Household composition		0.639	0.635
Single	0.06(1.0)		
Couple	0.01(1.0)		
3 people	-0.01(0.9)		
4-5 people	-0.05(0.9)		
6 people or more	0.01(0.8)		
Body Mass Index (BMI)		7.263	<0.001
Underweight	0.33(0.8)*°§		
Normal weight	0.03(0.9)*^		
Overweight	-0.14(1.1)°^		
Obese	-0.06(1.1)§		

Notes. Means scores and Standard Deviations (SD) of the HST index are reported. One-way ANOVA test was conducted with the psychological variables as independent factors and the HST as dependent factor. *F*, Fisher's coefficient. *,°,§, and ^, Between-groups means differences are significant ($p<0.05$) at multi comparisons LSD post-hoc test procedure.
